# The Effects of Vitamin E and Selenium on the Immune and Antioxidant Functions of Gushi Broiler Chickens After Immune Stress

**DOI:** 10.3390/ani16030462

**Published:** 2026-02-02

**Authors:** Sujin Si, Yixiang Tian, Xing Wu, Xuejie Ma, Yangguang Ren, Xiangtao Kang, Ruirui Jiang, Donghua Li, Yujie Gong, Yanhua Zhang, Yujie Guo, Yulong Guo, Yadong Tian

**Affiliations:** 1 College of Animal Science and Technology, Henan Agricultural University, Zhengzhou 450046, China; sisujin258@163.com (S.S.); t13071027821@163.com (Y.T.); wuxing6871@163.com (X.W.); maxuejie526@163.com (X.M.); jrrcaas@163.com (R.J.); lidonghua6656@126.com (D.L.); gongyj1118@163.com (Y.G.); YHzhang@henau.edu.cn (Y.Z.); 15093351679@163.com (Y.G.); sxauguoyulong@126.com (Y.G.); 2 Henan Key Laboratory for Innovation and Utilization of Chicken Germplasm Resources, Zhengzhou 450046, China

**Keywords:** broiler, selenium, Vitamin E, immunity, performance, antioxidant, lipopolysaccharide

## Abstract

This study investigated the effects of dietary supplementation with Vitamin E (**VE**) and selenium (**Se**) on immunostressed broilers. In the experiment, a total of 576 Gushi chickens were randomly allocated into 12 groups. They were respectively fed diets with different levels of VE and Se. Lipopolysaccharide (**LPS**) immune treatment was administered at 23, 25, and 27 days of age. At 28 days of age, the antioxidant indicators and immune factors in their serum, liver, and intestine were measured. The results indicated that (1) LPS treatment significantly elevated the contents of nitric oxide (**NO**) and nitric oxide synthase (**NOS**) in the duodenum and jejunum (*p* < 0.01). Conversely, adding VE and Se to the diet of LPS-treated chicks could significantly decrease the content of NO in the duodenum and the activities of NOS in the duodenum and jejunum (*p* < 0.01). (2) LPS treatment significantly decreased the content of malondialdehyde (**MDA**) in the liver of broilers, glutathione peroxidase (**GSH-P**x), and the activities of serum superoxide dismutase (**SOD**) (*p* < 0.01). Dietary supplementation of selenium could significantly reduce the content of MDA (*p* < 0.01), and the treatment groups with 200 mg/kg VE and 0.6 mg/kg Se exhibited the most favorable effects. (3) Adding Se or VE to the diet of LPS-treated chicks could significantly increase the thymus index and spleen index (*p* < 0.05) and significantly decrease the bursa of Fabricius index (*p* < 0.05). Among them, the thymus index was the highest in the 100 mg/kg VE group, and the interaction of the three factors had a significant influence on the thymus index and bursa of Fabricius index (*p* < 0.05). (4) The level ofinterleukin-6 (**IL-6**) in the serum of broilers in the LPS treatment group was significantly increased. However, selenium and Vitamin E were added to the diet of LPS-treated chicks to reduce the contents of serum IL-6 and IL-8, as well as adrenocorticotropic hormone (**ACTH**) and tumor necrosis factor-α (**TNF-α**). Moreover, the interaction of the three factors has a significant impact on immune indicators (*p* < 0.01). In conclusion, adding 100–200 mg/kg of VE and 0.6 mg/kg of Se to the diet of broiler chickens has certain advantages in enhancing immunity and antioxidant capacity.

## Simple Summary

Selenium is an essential trace element in the animal body, and its most important function is to participate in the composition of various selenoproteins in animals. Vitamin E is one of the most abundant fat-soluble antioxidants in the body and one of the important components of the body ’s antioxidant defense system. This study suggested that adding Vitamin E and selenium to the diet could alleviate the decline in immune and antioxidant capacity caused by immune stress in Gushi broilers. It had no significant promoting effect on the growth performance of Gushi broilers, but could enhance their immune and antioxidant functions.

## 1. Introduction

Currently, the global poultry farming scale continues to expand. Under the conditions of modern intensive production mode, the immune stress problem of Gushi broiler chickens has become increasingly prominent [1,2,3]. Immune stress can induce oxidative damage, impair immune function, and reduce the growth performance of Gushi broilers, thereby limiting the economic benefits of intensive poultry farming. In production, Gushi broiler chickens are often invaded by various pathogenic and non-pathogenic microorganisms, as well as stimulated by artificial immune antigens; these stress factors may also have an impact on the immune system of broiler chickens [4,5]. In chickens, immune stress can influence organ growth, proliferation of lymphocytes, percentages of CD4+ and CD8+, cytokine profiles, and the immune response of antigen to antibody [6,7,8]. Gao further confirmed that stress response can lead to a decline in poultry production performance and immune function, and even increase mortality rate, causing serious economic losses to breeding production [9,10].

At present, a large number of studies have found that, by optimizing the breeding environment parameters, standardizing the feeding management process, and conducting targeted genetic breeding, the probability of stress response in Gushi broiler chickens can be reduced, thereby alleviating the negative impact of stress on their growth performance and immune function. Due to factors such as fluctuations in environmental conditions, technical application costs, and facility barriers in actual breeding, reducing immune stress in Gushi broiler chickens through breeding environment is less commonly used in practical production. Therefore, regulating the antioxidant defense system of poultry through nutritional means to alleviate stress hazards has become a core focus of current research [11,12].

Se is an essential trace element in the animal body, and its most important function is to participate in the composition of various selenoproteins in animals, such as GSH-Px, which can remove oxidizing substances in the body, mediate antioxidation, and improve immune function [13,14]. VE is one of the most abundant fat-soluble antioxidants in the body and one of the important components of the body ’s antioxidant defense system [15]. In recent years, a number of studies have reported the use of Se and VE to reduce oxidative stress and improve immune performance in mammals [16,17,18], and similar results were found in aquatic animals [19,20]. In addition, studies have also found that adding Se and VE to the diet of poultry not only significantly improves their antioxidant capacity, but also effectively alleviates the adverse effects of immune stress on their growth performance [21,22,23].

Gushi broiler chicken is a well-known local breed in China, valued for its excellent meat quality. Bacterial lipopolysaccharide (LPS), also known as endotoxin, is currently a more effective immune stressors [24]. To the best of our knowledge, Gushi chicken has not been used as a model to study combinations of Se and VE or combinations of LPS, Se, and VE. Therefore, in this experiment, an immune stress model of broilers was constructed through an intraperitoneal injection of LPS, and VE and Se were added to the diet to explore their effects on the immune and antioxidant functions in LPS-challenged broilers. The results provide a theoretical foundation for alleviating immune stress in poultry production.

## 2. Materials and Methods

### 2.1. Experimental Birds and Management

In this study, commercial Gushi birds (*n* = 576) were reared in cages at a corporation in Xinyang for 28 d. The cage size was as follows: length, 0.6 m; width, 0.6 m; and height, 0.36 m. The stocking density was 30 birds between the ages of 1 and 9 d or 18 birds between the ages of 10 and 28 d in each cage. The temperature, humidity, light and ventilation were controlled. The basic diet was formulated according to the nutritional standard recommended by China ’s feeding standard (NY/T 33–2004) [25,26,27]. The composition and nutritional level of the basic diet are shown in Table 1.

### 2.2. Animal Grouping and Treatments

A total of 576 1 d old Gushi chickens were used and randomly divided into 12 treatments (3 Se levels × 4 VE levels) with 4 replicates per treatment and 12 chickens per replicate. A 3 × 4 completely random design was used in the experiment, and the chickens were fed the experimental diets with 0, 50, 100, or 200 mg/kg VE and 0, 0.3, or 0.6 mg/kg Se for 28 d.

In this study, 12 chickens in each replicate group were randomly divided into two groups (control group and stress group, *n* = 6). E. coli LPS was purchased from Sigma (Kawasaki, Japan, L2880). LPS was dissolved in normal saline and stored at 2–8 °C before injection. All the chicks in the experimental group (6 chicks per group, a total of 3 groups) were exposed to lipopolysaccharide (500 micrograms per kilogram body weight, intraperitoneal injection) stimulation before feeding at 23, 25, and 27 days of age [28,29,30].

The chickens belonging to the control group were injected with the same amount of normal saline.

Before sampling, the chickens were fasted for 12 h and not allowed to drink water for 2 h. Twenty-four hours after the third inoculation, one chicken from each replicate was randomly selected and weighed. Blood was then collected from the heart (anticoagulant), centrifuged at 1500 r/min for 10 min, placed in a 1.5 mL centrifuge tube, and stored at −20 °C. Liver tissue was removed, the tissue was homogenized at 0 °C and centrifuged at 3000 r/min for 15 min, and the supernatant was then collected. The intestines of the chickens were then separated, and the duodenum, jejunum, and ileum were collected. The length of the intestines, jejunum, and ileum was 5–6 cm. The intestinal contents were squeezed out (taking care not to damage the mucosa), placed in a 5 mL centrifuge tube, and stored at −20 °C. The immune organs (thymus, spleen, and bursal) were extracted, and the blood and water on the surface were dried with filter paper and then weighed.

### 2.3. Indicator Measurement

#### 2.3.1. Growth Performance

At the beginning of the experiment and at the start of each week, the flocks in each replicate were weighed, and the average body weight was calculated. Before weighing, the chickens were fasted for 12 h and not allowed to drink water for 2 h. Feed intake was assessed weekly. Each replicate was provided with feed in a separate, sealed container. At the end of each week, the remaining feed was weighed. The consumption by the chickens in each replicate group was accurately recorded to calculate the average daily feed intake, average daily gain, and feed-to-gain ratio.

#### 2.3.2. Antioxidant Index Detection

The activities of superoxide dismutase (SOD, A001-1-2), glutathione peroxidase (GSH-PX, A005-1-2), malondialdehyde (MDA, A003-1-2), nitric oxide (NO, A012-1-2) and NO synthase (NOS, A014-2-2) in the intestinal mucosa in the serum and liver of chickens were measured using kits purchased from Nanjing Jiancheng Bioengineering Institute (Nanjing, China).

#### 2.3.3. Estimation of Immune Parameters

The contents of ACTH, CD3+, CD4+, TNF-α, IL-1 β, and IL-6 in serum of the experimental chickens were determined using a GF-m2000 ELISA instrument from Shandong, Weifang, Gaomi (Shandong Huamei Analytical Instrument Co., Ltd.) and kits purchased from Nanjing Jiancheng Bioengineering Institute. The immune organ indexes were calculated as follows: immune organ index = immune organ weight (g)/body weight (kg).

### 2.4. Statistical Analysis

All data are presented as means ± SD. Growth performance variables (Table 2) were analyzed by a two-way ANOVA using the general linear model (GLM) in SPSS Statistics 20.0, with dietary selenium level and Vitamin E level as fixed factors, including their interaction (Se × VE). Data presented in Table 3, Table 4, Table 5 and Table 6 (antioxidant indices, intestinal NO/NOS, immune organ indices, and serum immune parameters) were analyzed by a three-way ANOVA (GLM, SPSS Statistics 20.0) with selenium level, Vitamin E level, and LPS challenge (saline vs. LPS) as fixed factors, including all two-way and three-way interactions (Se × VE, Se × LPS, VE × LPS, and Se × VE × LPS). Homogeneity of variance was checked using Levene’s test. When significant effects were detected, mean differences were assessed using Duncan’s multiple range test. Statistical significance was set at *p* < 0.05.

## 3. Results

### 3.1. The Effects of Se and VE on the Growth Performance of Gushi Chicken

As shown in Table 2, with the increase in Se and VE doses, the average daily weight gain of Gushi broiler chickens increased and the feed-to-meat ratio decreased, but the difference between groups was not significant. (*p* > 0.05). The groups fed diets supplemented with 100 mg/kg VE or 0.3 mg/kg Se exhibited the highest average daily gains and the lowest feed conversion ratios.

Dietary supplementation with Se, VE, or both Se and VE had no significant effects on the average daily feed intake, average daily gain, and feed-to-gain ratio of Gushi chickens (*p* > 0.05).

### 3.2. The Effects of Se, VE, and Immune Stress Treatment on the Antioxidant Capacity and Nitric Oxide Synthase of Gushi Broiler Chicken

As shown in Table 3 and Table 4, compared with the control group, LPS decreased the SOD content in serum and the GSH-Px and SOD contents in the liver (*p* < 0.01) and increased the MDA level in the liver (*p* < 0.01; Table 3). Supplementation with VE increased the GSH-Px and SOD contents in the liver (*p* < 0.01) and decreased the MDA level in serum and the liver (*p* < 0.01). Supplementation with Se increased the GSH-Px level in serum (*p* < 0.01) and the GSH-Px and SOD contents in the liver (*p* < 0.01) and decreased the MDA level in serum and the liver (*p* < 0.05). Further analysis of the interaction revealed that the interaction between VE and Se had a significant impact on the levels of GSH-Px, SOD, and MDA in serum. The interaction between VE and LPS treatment, Se and LPS treatment, VE, and Se and LPS had a significant effect on the levels of serum SOD, liver GSH Px, and SOD, but had no significant effect on the levels of serum GSH Px and MDA.

LPS increased the levels of NO and NOS in the duodenum and the level of NOS in the jejunum (*p* < 0.01) and decreased the NO level in the ileum (*p* < 0.01; Table 4). Se supplementation reduced the NO content in the duodenum (*p* < 0.01) and increased the NO content in the jejunum (*p* < 0.01). VE supplementation decreased the NO level in the ileum and the NOS level in the duodenum and jejunum (*p* < 0.01). Further analysis of the interaction revealed that the two-way interactions (Se × VE, Se × LPS, VE × LPS) and the three-way interaction (Se × VE × LPS) significantly affected the NO contents in the duodenum, jejunum, and ileum (*p* < 0.05). The interactions between Se and LPS, between VE and LPS, and among Se, VE, and LPS had no effect on the NOS contents in the jejunum and ileum (*p* > 0.05).

### 3.3. The Effects of Se, VE, and LPS Treatments on the Immune Organ Index of Gushi Broiler Chickens

Table 5 shows that, compared with the control group, the spleen index and bursal index of the immune stress treatment group were significantly increased (*p* < 0.01), while the thymus index was significantly decreased (*p* < 0.01). Supplementation of Vitamin E in the diet of chicks treated with LPS significantly increased the thymus index (*p* < 0.01) and significantly decreased the bursa of Fabricius index (*p* < 0.01), but had no effect on the spleen index (*p* > 0.05). The treatment with 100 mg/kg VE yielded the highest thymus index. Supplementation with Se significantly decreased the bursal index (*p* < 0.01), but had no effects on the spleen index or thymus index (*p* > 0.05). Further analysis of the interaction revealed that the interactions between VE and Se, between VE and LPS, between Se and LPS, and among VE, Se, and LPS had significant effects on the thymus and bursal indexes (*p* < 0.01).

### 3.4. The Effects of Se, VE, and LPS Treatment on Blood Biochemical Indicators in Gushi Broiler Chickens

As shown in Table 6, compared with the control group, LPS increased the content of IL-6 in serum (*p* < 0.05) and did not affect other indicators (*p* > 0.05). The Se treatments decreased the contents of ACTH, TNF-α, IL-1, and IL-6 (*p* < 0.01) and increased the levels of CD4+ and CD8+ (*p* < 0.01). The VE treatments decreased the contents of ACTH, TNF-α, and IL-6 (*p* < 0.01) and increased the level of CD4+ (*p* < 0.01). Further analysis of the interaction revealed that the interactions between VE and Se, between VE and LPS, between Se and LPS, and among VE, Se, and LPS had effects on ACTH, CD4+, CD8+, TNF-α, IL-1, and IL-6 contents (*p* < 0.05).

## 4. Discussion

### 4.1. The Influence of Se and VE on the Growth Performance of Broilers

Habibian et al. [31] found that the growth performance of broilers was not affected (*p* > 0.05) by VE (0, 125, and 250 mg/kg) and Se (0, 0.5, and 1 mg/kg) supplementation. Previous studies have revealed that hens fed different sources or different levels of Se (0.3–0.5 mg/kg) for 11 consecutive weeks exhibited no difference in their feed intake and feed conversion rate (*p* > 0.05) [32]. In this study, dietary supplementation with 0–200 mg/kg VE and 0–0.6 mg/kg Se had no effect on the body weight gain, average daily feed intake, or feed-to-gain ratio of broilers (*p* > 0.05), and this finding is in accordance with the results reported by other researchers [33]. Upton, J et al. [34] reported that 0.2 mg/k Se exerted an effect on the feed conversion rate of broilers (*p* < 0.05) but had no effect on the feed intake and daily gain (*p* > 0.05). In this study, dietary supplementation with 0–0.6 mg/kg Se had no effect on broiler performance, and chicks given supplementary Se (0.3 mg/kg) achieved their maximum body weight and optimal feed-to-gain ratio. The different effects of Se on performance might be related to the animal species, feedstuffs, environment, Se level, and sources, among other factors. However, the Se dose used in this study was higher than the broiler requirement (0.15 mg/kg) recommended by the NRC (1994). No adverse effects of a high dose of Se (not a moderate level) on the growth performance of broilers have been found in other studies [35,36]. Supplementary with VE at 100 mg/kg yielded the highest average daily gain.

### 4.2. The Effects of Se, VE and LPS Treatments on the Antioxidant Capacity of Gushi Broiler Chickens

The 0 to 28-day period is a crucial developmental window for Guoxi chickens: during this time, the intestinal structure of the chicks rapidly matures and the basic immune function gradually forms. In this early stage, chicks are particularly sensitive to the inflammatory response induced by lipopolysaccharide (LPS), so we focus here on the regulatory effects of selenium (Se) and Vitamin E (VE) on the antioxidant capacity of growing chicks.

GSH-Px and T-SOD are important enzymes in the enzymatic antioxidant system. Their activity is proportional to the body ’s ability to scavenge free radicals and reflects the body ’s antioxidant capacity. The levels of MDA are often used as an indication of oxidative damage and as a marker of free radical-induced lipid peroxidation [37]. VE and Se play important roles in protecting cells from reactive oxygen species (ROS) and in reducing the production of free radicals and lipid peroxides and are the most important antioxidants [38]. Some studies have found that dietary supplementation with Se and VE can improve blood parameter disorders and the oxidative stability of broilers under heat stress [38]. It has been reported that dietary Se and VE supplementation decreases the MDA content in breast meat (*p* < 0.05) under stress, and the lowest MDA content has been obtained with 1 mg/kg Se and VE [31]. LPS decreased the contents of SOD in serum and those of GSH-Px and SOD in liver (*p* < 0.01) and increased the MDA level in the liver (*p* < 0.01). Supplementation with VE increased the GSH-Px and SOD levels in the liver (*p* < 0.01) and decreased the MDA levels in serum and the liver (*p* < 0.01). Supplementation with Se increased the GSH-Px level in serum (*p* < 0.01) and the GSH-Px and SOD levels in the liver. This finding shows that LPS damages the body’s immune system and stimulates the oxidative damage activity of free radicals. Dietary supplementation with VE and Se reduces the MDA content, increases the SOD and GSH-PX activities, and relieves the immune stress response. Supplementation of VE and Se could decrease MDA content, increase SOD and GSH-PX activities, and alleviate immune stress reaction. The interaction between VE and Se had significant effects on the GSH-Px, SOD, and MDA levels in serum (*p* < 0.01). The interaction between VE and LPS, between Se and LPS, and among VE, Se, and LPS had significant effects on the SOD level in serum and the GSH-Px, SOD, and MDA contents in the liver (*p* < 0.05). The above results were consistent with previous studies. Coetzee and Hoffman [39] showed that the addition of 20–100 mg/kg VE to broiler diets can reduce the MDA content in broiler chicken products, and the MDA content decreased with increases in the level of VE. This finding is inconsistent with the results of the present study, and the difference may be related to the animal species, breeding environment, stress intensity, or other factors. The specific reasons will require further study.

Se is an important component of GSH-Px, and the activity of GSH-Px is closely related to the Se levels [40]. The addition of Se to the diet can increase the activity of GSH-Px in serum and the liver [41,42]. In this study, the activity of GSH-Px in serum and the liver was increased by the addition of Se. An Se dose of 0.6 mg/kg yielded the highest GSH-Px activity. Therefore, the specific reason for the increase in GSH-Px activity may be that dietary Se supplementation increases the body’s Se intake and blood Se level and thus causes an increase in plasma GSH-Px activity. In this study, the serum SOD activity of broilers first increased and then decreased with increases in the Se content in the diet. The highest SOD activity was obtained with the treatment with 0.30 mg/kg Se. The addition of Se at a dose of 0.60 mg/kg decreased the SOD activity, which indicated that Se at a dose within a certain range exerts a protective effect on broilers. In addition, MDA content in serum and liver was negatively correlated with GSH-Px activity, and the interaction of VE and Se significantly increased GSH-Px activity (*p* < 0.05), which confirmed that VE and Se had synergistic antioxidant effect and could effectively relieve immune stress.

The intestinal tract is closely related to poultry stress. LPS challenge can lead to injury of the intestinal mucosa and an increase in intestinal permeability, which can lead to an inflammatory reaction [43,44]. NO is a neurotransmitter in the nervous system that mediates neurophysiological and pathological processes by participating in information transmission. The catalytic enzyme NOS is a lipophilic gas free radical that can penetrate into the biofilm and act as a messenger between and within cells [45,46].

Mishima et al. [47] found that increases in NO production and NOS activity could lead to intestinal mucosal damage and bacterial translocation in the intestine. In this study, LPS increased the NO and NOS level in the duodenum and the NOS levels in the jejunum (*p* < 0.01) and decreased the NO levels in the ileum (*p* < 0.01). Se and VE supplementation decreased the NO levels in the duodenum and ileum and the NOS levels in the duodenum and jejunum (*p* < 0.01). The results suggest that Se and VE can alleviate intestinal injury after immune stress. Se supplementation may inhibit excessive NOS activation and NO production by enhancing the antioxidant capacity of intestinal mucosa, thereby alleviating LPS-induced intestinal damage. Supplementation with VE promotes the repair of tissue mucosal damage and supports the integrity of intestinal mucosa cells via contact, which results in reducing the degree of intestinal mucosa damage.

Further analysis of the differences in different intestinal segments showed that dietary Se 0~ 0.6 mg/kg and VE 0~200 mg/kg had no significant effect on NOS level in ileum, which may be due to jejunum being the main part of nutrient absorption and preferential utilization of VE in chyme, resulting in insufficient VE dose reaching ileum and unable to induce NOS-level change; a high dose of VE (100~200 mg/kg) combined with Se (0. 6 mg/kg) could decrease NOS activity in duodenum and jejunum and NO content in ileum to the minimum, indicating that a high dose of VE and Se could strengthen intestinal barrier function by inhibiting excessive production of NO and NOS in intestine.

### 4.3. Effects of Se, VE and LPS on Immune Organ Index of Gushi Broilers

The thymus, spleen, and bursal are important immune organs for poultry because they can resist the invasion of pathogens; these organs are easily affected by immune stimulation. The weight of immune organs can be used to measure the immune status of poultry [48]. Some researchers have determined that stress could cause atrophy of the thymus and bursal [49]. In this experiment, LPS challenge increased the spleen index (*p* < 0.01) and decreased the thymus and bursal indexes (*p* < 0.01). These changes may have been obtained because the proliferation of lymphocytes in the spleen under immune stress leads to enhance immune resistance and splenomegaly. Hegazy and Adachi [50] reported that supplementation with Se alone or in combination with VE significantly increases the average weight gain of the lymphatic organs (bursal, spleen, and thymus) of broiler chickens compared with that of the control group. Van den Berg et al. [51] showed that dietary supplementation with VE increased the weights of the thymus and bursal (*p* < 0.05). In our study, supplementation with VE increased the thymus index and decreased the bursal index (*p* < 0.01). The highest thymus index was obtained with 100 mg/kg VE. The interaction between VE and LPS also had a very significant effect on the thymus index (*p* < 0.01). This finding is inconsistent with previous results, and the difference may be related to the test animal species, breeding environment, and immune strength, but the specific reasons need to be further studied.

It is generally believed that an increase in the immune organ index is a manifestation of immune enhancement and that a decrease in the immune organ index is a result of immune suppression [52]. Some researchers have determined that the addition of Se increases the immune organ index of broilers. In this study, dietary Se supplementation had no effect on the spleen and thymus indexes (*p* > 0.05). Dietary supplementation with VE or Se decreased the bursal index (*p* < 0.01). The immune stress may have caused the bursal of Fabricius to be too seriously damaged such that supplementation with VE and Se could not induce an improvement, and this finding may be related to the intensity of stress. The specific reasons remain to be further discussed.

### 4.4. Effects of Selenium, Vitamin E, and LPS on Blood Biochemical Parameters in Gushi Broilers

Cytokines (CKs) are small-molecule polypeptides produced by a variety of cells that can regulate cell growth, differentiation, and immune function. Cytokines specifically include more than a dozen types of IFN-α, IFN-β, IL, and TNF-α. Some researchers have determined that LPS can induce the production of proinflammatory mediators and proinflammatory cytokines such as TNF-α, IL-6, and IL-1 β [53]. In this study, LPS increased the content of IL-6 (*p* < 0.05) and had no significant effects on other factors (*p* > 0.05). The immune stress induced by LPS may lead to abnormal changes in blood biochemical indicators that can be maintained for only a short period of time (1–6 h), and although complex immune procedures can cause immune stress, the intensity is not sufficient to cause high-intensity oxidation in the body [54,55]. Studies have found that immune stress increases the ACTH level in blood. In this study, LPS had no effect on the ACTH level, which may depend on the nature of the stressor, the intensity of the stimulation, or the duration of the stimulation [56].

Dietary supplementation with VE can improve the immune function of broilers [57,58]. It has been reported that dietary supplementation with VE can reduce the secretion of IL-6, IL-1 β, and TNF-a in LPS-challenged rats and has no effect on IL-2 [59,60]. Some studies have shown that dietary supplementation with 0.2 mg/kg Se and 200 mg/kg VE increases the antibody level of broilers (*p* < 0.05). Studies have found that supplementation with Se can reduce the levels of IL-2 and TNF-α in normal mouse serum in a dose-dependent manner (*p* < 0.05). In this study, supplementation with VE or Se reduced the ACTH, TNF-α, and IL-6 contents (*p* < 0.01). The lowest concentrations of TNF-α, IL-6, and ACTH were obtained with 0.6 mg/kg Se supplementation. Additionally, the lowest concentrations of TNF-α and IL-6 were obtained with 100 mg/kg VE supplementation, and the interaction between VE and Se had an effect on these serum indexes (*p* < 0.01). These results indicate that VE and Se can antagonize or partially antagonize the changes in cytokine levels caused by LPS and also show that different cytokines exhibit different sensitivities to VE and Se. The synthesis and secretion of cytokines is a self-regulating process; the antioxidant system in the body interacts with cytokines to form a cytokine network [61].

The CD4+-to-CD8+ ratio is an important indicator of immune suppression, and a decrease in the CD4+-to-CD8+ ratio is observed after stress. In this study, 100–200 mg/kg VE supplementation did not affect serum CD8+ levels but significantly increased CD4+ contents, thereby elevating the CD4+/CD8+ ratio—a key marker of improved immune function. These results show that VE can improve the immune function of broiler chickens [62]. In this study, with increases in the dose of VE, the level of TNF-α first decreased and then increased. One possible reason is that the lipid oxidation process in animals is mediated mainly by TNF-α. During immunological stress, the intake decreases and lipid oxidation increases to provide energy, and this finding is consistent with the results reported by Zachut et al. [63].

## 5. Conclusions

In conclusion, the addition of VE and Se to the diet can alleviate the decline in immune and antioxidant capacity caused by immune stress in Gushi broilers. The most significant effect is achieved when 0.6 mg/kg Se and 100–200 mg/kg VE are added. It does not significantly promote the growth performance of Gushi broilers, but it can enhance their immune and antioxidant functions.

## Figures and Tables

**Table 1:** Ingredients and nutrient composition of the experimental basal diet (% as fed).

Item	Proportion of Ingredients
Corn	62.40
Soybean meal	30.00
Fish meal	3.00
Dicalcium phosphate	1.30
Limestone	1.30
Salt	0.20
*L*-Methionine (98%)	0.20
Soybean oil	0.60
Premix ^1^	1.00
**Nutrients**	
Metabolite energy (MJ/kg)	12.00
Crude protein (%)	20.36
Methionine (%)	0.53
Methionine + Cysteine (%)	0.80
Lysine (%)	1.06
Ca (%)	1.10
AP (%)	0.46
VE (mg/kg)	6.67
Se (mg/kg)	0.08

^1^ Added to each group of test diets by premix (content per kg of food): VA 10,000 IU, VD 32,500 IU, VK 33 mg, VB 13 mg, VB2 5.5 mg, VB 61 mg, VB12 0.01 mg, niacin 34 mg, calcium pantothenate 12 mg, folic acid 0.5 mg, organism 0.2 mg, choline 400 mg, Fe 22.5 mg, Cu 60 mg, Zn 48.75 mg, Mn 75 mg, I 0.75 mg, antibiotics, antioxidants, etc. ME was the calculated value and the others represent the measured value.

**Table 2:** Effect of dietary supplementation with Selenium and Vitamin E on the performance of 28-day-old Gushi chickens.

Group		Average Daily Feed Intake (g/d)	Average Daily Gain (g/d)	Feed-to-Gain Ratio
Selenium (mg/kg)	Vitamin E (mg/kg)			
0		35.28 ± 3.58	17.48 ± 0.87	2.02 ± 0.20
0.3		34.30 ± 1.98	17.82 ± 1.15	1.93 ± 0.16
0.6		34.90 ± 2.90	17.66 ± 1.26	1.99 ± 0.24
	0	33.92 ± 1.94	17.60 ± 1.00	1.93 ± 0.16
	50	35.61 ± 3.75	17.67 ± 1.13	2.02 ± 0.24
	100	35.02 ± 2.2	17.75 ± 1.02	1.95 ± 0.17
	200	34.75 ± 3.26	17.59 ± 1.32	1.96 ± 0.24
*p*-value	Selenium	0.646	0.707	0.455
	Vitamin E	0.575	0.984	0.759
	Selenium × Vitamin E	0.564	0.316	0.215

**Table 3:** Effect of dietary Selenium and Vitamin E on antioxidant parameters in the blood and liver of 28-day-old Gushi chickens exposed to LPS.

Group			Serum GSH-Px (nmol/L)	Serum SOD (nmol/L)	Serum MDA (nmol/L)	Liver GSH-PX (nmol/L)	Liver SOD (nmol/L)	Liver MDA (nmol/L)
Selenium (mg/kg)	Vitamin E (mg/kg)	LPS						
0			1430 ± 95.41 ^b^	57.28 ± 4.37	243.33 ± 21.24 ^a^	96.70 ± 40.39 ^c^	405.31 ± 118.03 ^b^	5.24 ± 5.59 ^a^
0.3			1970 ± 92.16 ^a^	58.07 ± 8.88	228.33 ± 24.3 ^ab^	138.09 ± 57.54 ^b^	425.39 ± 132.67 ^b^	3.97 ± 1.83 ^b^
0.6			1962.2 ± 90.44 ^a^	55.97 ± 13.40	213.33 ± 21.92 ^b^	174.15 ± 55.47 ^a^	467.51 ± 183.27 ^a^	3.37 ± 1.38 ^b^
	0		1788.57 ± 84.01	61.56 ± 15.40 ^a^	296.67 ± 34.43	127.49 ± 59.69 ^c^	469.62 ± 142.97 ^b^	7.46 ± 5.61 ^a^
	50		1792.86 ± 87.01	59.90 ± 8.39 ^a^	264.44 ± 30.95	121.15 ± 44.24 ^c^	422.86 ± 123.54 ^b^	2.67 ± 1.34 ^b^
	100		1794.83 ± 84.12	56.04 ± 6.44 ^b^	241.11 ± 36.15	142.00 ± 61.12 ^b^	403.16 ± 164.97 ^b^	3.84 ± 1.31 ^b^
	200		1792 ± 85.42	53.28 ± 8.35 ^b^	239.11 ± 32.35	154.6 ± 69.04 ^a^	471.66 ± 155.75 ^a^	2.82 ± 1.06 ^b^
		0	1798.93 ± 95.11	59.30 ± 12.85 ^a^	245.83 ± 52.82	170.79 ± 58.50 ^a^	529.75 ± 140.19 ^a^	3.35 ± 1.41 ^b^
		1	1758.37 ± 93.26	55.81 ± 7.50 ^b^	273.61 ± 51.72	101.84 ± 39.35 ^b^	335.73 ± 79.26 ^b^	5.04 ± 4.70 ^a^
*p*-value		Selenium	<0.001	0.867	0.029	<0.001	<0.001	<0.001
		Vitamin E	0.318	<0.001	0.242	<0.001	0.002	<0.001
		LPS	0.630	0.004	0.139	<0.001	<0.001	<0.001
		Selenium × Vitamin E	0.001	<0.001	<0.001	<0.001	<0.001	<0.001
		Selenium × LPS	0.573	0.003	0.829	0.011	<0.001	<0.001
		Vitamin E × LPS	0.812	<0.001	0.726	<0.001	0.035	<0.001
		Selenium × Vitamin E × LPS	0.472	<0.001	0.732	<0.001	<0.001	<0.001

Means in the same columns with different superscripts differ significantly (*p* < 0.05), *n* = 6.

**Table 4:** Effects of Selenium and Vitamin E on nitric oxide and nitric oxide synthase in the intestinal tract of 28-day-old Gushi chickens exposed to LPS.

Group			Duodenal NO (μmol/g prot)	Jejunal NO (μmol/g prot)	Ileal NO (μmol/g prot)	Duodenal NO Synthase (U/mg prot)	Jejunal NO Synthase (U/mg prot)	Ileal NO Synthase (U/mg prot)
Selenium (mg/kg)	Vitamin E (mg/kg)	LPS						
0			0.065 ± 0.027 ^a^	0.18 ± 0.05 ^b^	0.07 ± 0.02 ^a^	3.54 ± 0.83 ^a^	8.1 ± 1.06 ^a^	1.92 ± 0.31
0.3			0.058 ± 0.006 ^c^	0.28 ± 0.11 ^a^	0.07 ± 0.03 ^a^	2.84 ± 0.62 ^b^	7.22 ± 0.45 ^b^	1.79 ± 0.24
0.6			0.061 ± 0.024 ^b^	0.29 ± 0.11 ^a^	0.04 ± 0.01 ^b^	2.35 ± 0.62 ^c^	6.87 ± 0.42 ^c^	1.83 ± 0.1
	0		0.045 ± 0.010 ^b^	0.18 ± 0.07 ^c^	0.068 ± 0.030 ^a^	3.31 ± 0.81 ^a^	7.85 ± 0.9 ^a^	2.00 ± 0.29
	50		0.071 ± 0.028 ^c^	0.29 ± 0.10 ^a^	0.068 ± 0.023 ^a^	3 ± 0.87 ^b^	7.76 ± 0.97 ^a^	1.88 ± 0.21
	100		0.078 ± 0.010 ^a^	0.29 ± 0.08 ^a^	0.058 ± 0.014 ^b^	2.76 ± 0.84 ^ab^	7.15 ± 0.62 ^c^	1.77 ± 0.22
	200		0.052 ± 0.011 ^b^	0.24 ± 0.08 ^b^	0.056 ± 0.025 ^b^	2.58 ± 0.76 ^c^	7.23 ± 0.51 ^b^	1.74 ± 0.09
		0	0.060 ± 0.025 ^b^	0.25 ± 0.13	0.21 ± 0.33 ^a^	2.58 ± 0.69 ^b^	7.10± 0.76 ^b^	1.81 ± 0.21
		1	0.064 ± 0.015 ^a^	0.25 ± 0.08	0.06 ± 0.02 ^b^	3.24 ± 0.87 ^a^	7.69 ± 0.87 ^a^	1.88 ± 0.26
*p*-value		Selenium	<0.001	<0.001	<0.001	<0.001	<0.001	0.187
		Vitamin E	<0.001	0.008	<0.001	<0.001	<0.001	0.187
		LPS	<0.001	0.77	<0.001	0.001	0.003	0.189
		Selenium × Vitamin E	<0.001	0.042	<0.001	0.850	<0.001	0.780
		Selenium × LPS	<0.001	0.001	<0.001	0.785	0.120	0.987
		Vitamin E × LPS	0.003	0.019	<0.001	0.002	0.972	0.976
		Selenium × Vitamin E × LPS	<0.001	0.023	<0.001	<0.001	0.784	0.986

Means in the same columns with different superscripts differ significantly (*p* < 0.05), *n* = 6.

**Table 5:** Effect of Selenium and Vitamin E on the immune organ index of 28-day-old Gushi chickens exposed to LPS.

Group			Spleen Index	Thymus Index	Bursal Index
Selenium (mg/kg)	Vitamin E (mg/kg)	LPS			
0			1.94 ± 0.28	4.67 ± 0.88	3.06 ± 0.52 ^a^
0.3			2.07 ± 0.33	4.67 ± 0.72	2.71 ± 0.48 ^b^
0.6			2.00 ± 0.42	4.51 ± 0.70	2.34 ± 0.50 ^c^
	0		1.95 ± 0.50	4.42 ± 0.53 ^bc^	2.74 ± 0.54 ^ab^
	50		2.09 ± 0.30	4.24 ± 0.53 ^c^	2.99 ± 0.68 ^a^
	100		1.94 ± 0.25	4.97 ± 0.98 ^a^	2.55 ± 0.56 ^b^
	200		1.98 ± 0.32	4.83 ± 0.73 ^ab^	2.53 ± 0.37 ^b^
		0	1.82 ± 0.27 ^b^	4.75 ± 0.77 ^a^	2.46 ± 1.17 ^b^
		1	2.19 ± 0.32 ^a^	4.48 ± 0.75 ^b^	2.72 ± 0.48 ^a^
		Selenium	0.614	0.197	<0.001
		Vitamin E	0.718	<0.001	<0.001
		LPS	0.001	0.002	<0.001
*p*-value		Selenium × Vitamin E	0.132	<0.001	<0.001
		Selenium × LPS	0.774	<0.001	<0.001
		Vitamin E × LPS	0.116	<0.001	<0.001
		Selenium × Vitamin E × LPS	0.813	<0.001	<0.001

Means in the same columns with different superscripts differ significantly (*p* < 0.05), *n* = 6.

**Table 6:** Effects of Selenium and Vitamin E on serum immune parameters of 28-day-old Gushi chickens exposed to LPS.

Group			ACTH (ng/L)	CD4^+^ (U/mL)	CD8^+^ (U/mL)	TNF-α (U/mL)	IL-1 (U/mL)	IL-6 (U/mL)
Selenium (mg/kg)	Vitamin E (mg/kg)	LPS						
0			324.66 ± 20.05 ^a^	728.29 ± 27.3 ^c^	864.72 ± 73.87 ^a^	253.32 ± 16.39 ^a^	20.99 ± 2.78 ^c^	41.98 ± 2.01 ^a^
0.3			311.26 ± 24.21 ^b^	754.72 ± 23.27 ^b^	879.12 ± 69.56 ^b^	232.24 ± 10.96 ^b^	16.72 ± 2.58 ^b^	40.3 ± 2.82 ^b^
0.6			305.27 ± 24.98 ^b^	1190.55 ± 61.72 ^a^	899.72 ± 74.77 ^c^	215.59 ± 15.55 ^c^	14.69 ± 3.73 ^a^	38.96 ± 3.12 ^c^
	0		300.63 ± 68.94 ^a^	688.4 ± 36.68 ^c^	861.34 ± 35.1	281.97 ± 10.75 ^a^	17.6 ± 4.57	43.56 ± 2.53 ^a^
	50		278.1 ± 75.13 ^b^	889.38 ± 72.96 ^b^	865.66 ± 82.91	249.45 ± 10.23 ^b^	17.14 ± 2.56	40.38 ± 3.22 ^b^
	100		267.08 ± 12.14 ^c^	948.53 ± 72.52 ^a^	887.93 ± 52.69	191.11 ± 15.07 ^c^	14.79 ± 4.72	36.42 ± 3.91 ^c^
	200		257.11 ± 44.95 ^d^	938.43 ± 86.71 ^a^	860.83 ± 63.57	242.35 ± 18.54 ^b^	14.33 ± 2.44	43.63 ± 2.11 ^a^
		0	335.68 ± 97.51	935.85 ± 61.46	855.68 ± 59.01	243.53 ± 17.03	17.52 ± 3.84	42.15 ± 4.56 ^b^
		1	315.77 ± 25.14	926.52 ± 10.56	831.7 ± 85.22	243.91 ± 16.85	17.81 ± 2.24	45.35 ± 2.31 ^a^
		Selenium	<0.001	<0.001	<0.001	<0.001	<0.001	<0.001
		Vitamin E	<0.001	<0.001	0.070	<0.001	0.100	<0.001
		LPS	0.454	0.444	0.144	0.087	0.912	0.036
*p*-value		Selenium × Vitamin E	<0.001	<0.001	<0.001	<0.001	0.001	<0.001
		Selenium × LPS	<0.001	<0.001	<0.001	<0.001	0.002	<0.001
		Vitamin E × LPS	<0.001	<0.001	<0.001	<0.001	0.036	<0.001
		Selenium × Vitamin E × LPS	<0.001	<0.001	<0.001	<0.001	0.016	<0.001

Means in the same columns with different superscripts differ significantly (*p* < 0.05), *n* = 6.

## Data Availability

The original contributions presented in the study are included in the article/Supplementary Material, further inquiries can be directed to the author.

## Supplementary Materials

The following supporting information can be downloaded at: https://www.mdpi.com/article/10.3390/ani16030462/s1, Table S1. Effect of dietary Selenium and Vitamin E on antioxidant parameters in the blood and liver of 28-day-old Gushi chickens exposed to LPS. Table S2. Effects of Selenium and Vitamin E on nitric oxide and nitric oxide synthase in the intestinal tract of 28-day-old Gushi chickens exposed to LPS. Table S3. Effect of Selenium and Vitamin E on the immune organ index of 28-day-old Gushi chickens exposed to LPS. Table S4. Effects of Selenium and Vitamin E on serum immune parameters of 28-day-old Gushi chickens exposed to LPS.
