# Supplementary material for: The Effects of Vitamin E and Selenium on the Immune and Antioxidant Functions of Gushi Broiler Chickens After Immune Stress
Source: Animals (Basel). 2026 Feb 2;16(3):462. doi: 10.3390/ani16030462 (PMC12897321; doi:10.3390/ani16030462)
Supplement: Supplementary file 1 [file animals-16-00462-s001.zip › animals-4056168-supplementary.pdf]

Table S1. Effect of dietary Selenium and Vitamin E on antioxidant parameters in the blood and liver of 28-day-old Gushi chickens exposed to LPS.

Group			Serum GSH-Px(nmol/L)	Serum SOD (nmol/L)	Serum MDA(nmol/L)	Liver GSH-PX(nmol/L)	Liver SOD (nmol/L)	Liver MDA (nmol/L)
Selenium (mg/kg)	Vitamin E (mg/kg)	LPS						
0	0	0	1422.86 ± 54.24	53.54 ± 1.31	133.33 ± 21.10	124.8 ± 5.70	513.6 ± 12.83	19.47 ± 0.41
		1	1434.29 ± 61	55.38 ± 5.22	120 ± 10.00	67.45 ± 9.05	320.18 ± 12.50	5.20± 0.43
	50	0	1588.57 ± 98.97	52.92 ± 1.76	173.33 ± 13.09	92.60 ± 0.31	544.89 ± 43.14	2.38 ±0.29
		1	1154.29 ± 91.99	59.95 ± 0.69	240 ± 12.00	56.3 ± 6.92	272.11 ± 17.16	1.56 ± 0.03
	100	0	1514.28 ± 71.23	60.92 ± 1.31	266.67 ± 10.66	180.14 ± 10.19	536.81 ± 58.86	5.51 ± 1.00
		1	1400 ± 71.72	55.77 ± 2.89	266.67 ± 23.27	106.62±26.35	238.90 ± 34.86	3.93 ± 0.12
	200	0	1531.43 ± 54.46	59.69 ± 5.25	333.33 ± 21.1	90.15 ± 0.82	370.58 ± 8.38	2.39 ± 0.06
		1	1394.29 ± 94.01	43.08 ± 6.57	333.33 ± 23.1	55.56 ± 0.90	445.42 ± 14.57	1.48 ± 0.00
0.3	0	0	1965.71 ± 35.68	49.85 ± 2.63	320 ± 29.28	224.87 ± 6.07	720.85 ± 57.17	7.64 ± 0.45
		1	1817.14 ± 80.61	58.46 ± 0.87	360 ± 24.22	58.68 ± 4.38	446.96 ± 41.05	2.57 ± 0.16
	50	0	1685.71 ± 80.07	70.15 ± 5.24	306.67 ± 31.10	162.11 ± 15.95	460.24 ± 3.89	5.42± 0.42
		1	1931.43 ± 62.03	65.23 ± 1.78	320 ± 30.00	111.4 ± 32.13	380.56 ± 62.06	2.82 ± 0.20
	100	0	2074.29 ± 66.13	62.71 ± 1.36	120 ± 0.00	100.3 ± 21.75	305.67 ± 50.27	19.30± 0.50
		1	2342.86 ± 53.3	44.31 ± 2.64	200 ± 10.00	94.38 ± 8.62	276.65 ± 11.42	4.19 ± 0.16
	200	0	2137.14 ± 81.15	51.94 ± 5.77	226.67 ± 61.10	210.82 ± 23.63	390.77 ± 21.00	2.08 ± 0.24
		1	1805.71 ± 19.12	59.69 ± 4.79	253.33 ± 23.09	142.11 ± 16.39	421.44 ± 15.26	4.77± 0.33
0.6	0	0	1994.29 ± 37.75	93.74 ± 3.19	293.33 ± 18.58	181.75 ± 5.35	512.39 ± 11.65	3.95 ± 0.00
		1	2097.14 ± 93.29	55.38 ± 1.19	253.33 ± 10.66	107.41 ± 1.38	303.76 ± 2.06	5.90 ± 0.08
	50	0	2085.71 ± 42.64	48.00 ± 5.23	400 ± 30.00	177.82 ± 18.09	574.59 ± 84.35	1.63 ± 0.48

	1	1771.43 ± 57.08	60.31 ± 6.10	386.67 ± 31.10	126.69 ± 6.86	304.77 ± 25.90	2.18 ± 0.0.04
100	0	1131.43 ± 80.65	57.23 ± 3.55	173.33 ± 21.10	257.15 ± 15.22	680.24 ± 78.16	4.78 ± 0.26
	1	1166.14 ± 54.29	56.61 ± 0.89	240 ± 10.00	113.43 ± 7.06	380.75 ± 46.95	2.38 ± 0.82
200	0	1165.71 ± 90.71	48.62 ± 5.68	253.33 ± 10.66	246.98 ± 34.69	746.36 ± 45.04	2.96 ± 0.00
	1	1485.71 ± 40.42	41.23 ± 2.63	346.67 ± 26.19	182.00 ± 1.98	237.23 ± 9.97	3.23 ± 0.00

Each Value represents mean ± standard error (SE) of 6 individual broilers.

Table S2. Effects of Selenium and Vitamin E on nitric oxide and nitric oxide synthase in the intestinal tract of 28-day-old Gushi chickens exposed to LPS

Group			Duodenal NO (μmol/g prot)	Jejunal NO (μmol/g prot)	Ileal NO (μmol/g prot)	Duodenal NO synthase (U/mg prot)	Jejunal NO synthase (U/mg prot)	Ileal NO synthase (U/mg prot)
Selenium (mg/kg)	Vitamin E (mg/kg)	LPS						
0	0	0	0.03 ± 0.00	0.10 ± 0.00	0.05 ± 0.00	4.85 ± 0.2	7.66 ± 1.29	1.92 ± 0.07
		1	0.04 ± 0.00	0.16 ± 0.00	0.06 ± 0.00	4.83 ± 0.31	9.27 ± 0.29	2.18 ± 0.61
	50	0	0.11 ± 0.01	0.22 ± 0.01	0.06 ± 0.00	3.26 ± 0.39	7.6 ± 0.71	1.65 ± 0.02
		1	0.07 ± 0.00	0.27 ± 0.06	0.09 ± 0.01	4.57 ± 0.36	9.13 ± 1.23	2.02 ± 0.34
	100	0	0.08 ± 0.00	0.18 ± 0.01	0.06 ± 0.00	3.43 ± 0.21	6.02 ± 0.42	0.97 ± 0.33
		1	0.08 ± 0.00	0.19 ± 0.01	0.08 ± 0.00	3.62 ± 0.25	8.22 ± 0.44	1.83 ± 0.17
	200	0	0.05 ± 0.00	0.14 ± 0.00	0.09 ± 0.01	2.03 ± 0.43	5.66 ± 0.27	1.54 ± 0.39
		1	0.04 ± 0.00	0.16 ± 0.00	0.06 ± 0.00	2.68 ± 0.58	7.5 ± 0.15	1.77 ± 0.07
0.3	0	0	0.05 ± 0.00	0.18 ± 0.04	0.13 ± 0.01	2.31 ± 0.33	7.78 ± 0.42	1.32 ± 0.57
		1	0.06 ± 0.00	0.16 ± 0.00	0.06 ± 0.00	3.78 ± 0.17	7.91 ± 0.54	2.05 ± 0.36
	50	0	0.06 ± 0.00	0.20 ± 0.00	0.10 ± 0.01	2.04 ± 0.74	7.66 ± 1.17	1.78 ± 0.14
		1	0.06 ± 0.00	0.25 ± 0.01	0.07 ± 0.01	3.27 ± 0.32	7.6 ± 0.3	1.81 ± 0.13
	100	0	0.06 ± 0.00	0.31 ± 0.03	0.06 ± 0.01	1.54 ± 0.07	5.63 ± 0.13	1.23 ± 0.11
		1	0.07 ± 0.00	0.32 ± 0.01	0.04 ± 0.00	2.96 ± 0.25	7.27 ± 0.4	1.76 ± 0.38
	200	0	0.06 ± 0.00	0.51 ± 0.07	0.07 ± 0.01	1.55 ± 0.07	5.21 ± 1.06	1.71 ± 0.25
		1	0.05 ± 0.00	0.33 ± 0.00	0.05 ± 0.00	2.63 ± 0.31	7.18 ± 0.22	1.72 ± 0.1
0.6	0	0	0.04 ± 0.00	0.2 ± 0.06	0.05 ± 0.00	1.62 ± 0.14	7.99 ± 0.8	0.71 ± 0.05
		1	0.05 ± 0.00	0.3 ± 0.01	0.06 ± 0.01	2.86 ± 0.2	7.21 ± 0.16	1.92 ± 0.03

50	0	0.03 ± 0.00	0.42 ± 0.09	0.04 ± 0.00	2.28 ± 0.25	4.97 ± 1.86	0.98 ± 0.12
	1	0.09 ± 0.00	0.4 ± 0.07	0.04 ± 0.00	2.75 ± 0.49	7.34 ± 0.32	1.88 ± 0.03
100	0	0.09 ± 0.00	0.36 ± 0.08	0.05 ± 0.01	1.00± 0.05	4.95 ± 0.26	0.88 ± 0.1
	1	0.08 ± 0.00	0.34 ± 0.03	0.05 ± 0.00	2.58 ± 1.15	6.92 ± 0.06	1.83 ± 0.14
200	0	0.03 ± 0.00	0.16 ± 0.02	0.02 ± 0.00	1.25 ± 0.2	5.22 ± 0.32	0.55 ± 0.05
	1	0.06 ± 0.00	0.16 ± 0.01	0.03 ± 0.00	2.37 ± 0.3	6.78 ± 0.37	1.82 ± 0.02

Means in a row with different superscripts differ significantly ($P < 0.05$).

Each Value represents mean ± standard error (SE) of 3 individual broilers.

Table S3. Effect of Selenium and Vitamin E on the immune organ index of 28-day-old Gushi chickens exposed to LPS

Group			Spleen index	Thymus index	Bursal index
Selenium (mg/kg)	Vitamin E (mg/kg)	LPS			
0	0	0	1.60 ± 0.15	4.37 ± 0.05	3.56 ± 0.01
		1	2.34 ± 0.13	4.51 ± 0.36	2.53 ± 0.03
	50	0	1.78 ± 0.16	3.38 ± 0.23	4.15 ± 0.04
		1	1.83 ± 0.07	3.94 ± 0.16	3.08 ± 0.16
	100	0	1.88 ± 0.15	4.59 ± 0.14	2.84 ± 0.06
		1	2.01 ± 0.01	6.21 ± 0.45	2.90 ± 0.04
	200	0	2.07 ± 0.04	5.22 ± 0.35	2.53 ± 0.20
		1	2.14 ± 0.05	5.09 ± 0.65	2.90 ± 0.11
0.3	0	0	1.52 ± 0.13	3.89 ± 0.34	2.07 ± 0.24
		1	1.99 ± 0.06	4.69 ± 0.15	3.23 ± 0.12
	50	0	2.01 ± 0.06	4.24 ± 0.17	3.07 ± 0.10
		1	2.54 ± 0.05	4.92 ± 0.03	2.12 ± 0.09
	100	0	1.71 ± 0.01	6.27 ± 0.17	2.33 ± 0.05
		1	2.16 ± 0.33	3.94 ± 0.03	3.40 ± 0.21
	200	0	2.27 ± 0.04	4.77 ± 0.07	2.48 ± 0.03
		1	2.36 ± 0.14	4.61 ± 0.04	2.31 ± 0.30
0.6	0	0	1.57 ± 0.13	5.04 ± 0.28	2.37 ± 0.23
		1	2.78 ± 0.11	4.00 ± 0.62	2.02 ± 0.12

50	0	2.02 ± 0.10	4.71 ± 0.23	3.20 ± 0.15
	1	2.38 ± 0.18	4.25 ± 0.05	2.33 ± 0.32
100	0	1.91 ± 0.20	4.81 ± 0.08	2.12 ± 0.02
	1	1.98 ± 0.30	3.99 ± 0.30	1.73 ± 0.16
200	0	1.52 ± 0.02	5.69 ± 0.28	2.97 ± 0.05
	1	1.84 ± 0.08	3.58 ± 0.07	2.00 ± 0.15

Each Value represents mean ± standard error (SE) of 6 individual broilers.

Table S4. Effects of Selenium and Vitamin E on serum immune parameters of 28-day-old Gushi chickens exposed to LPS

Group			ACTH (ng/L)	$CD4^+$ (U/mL)	$CD8^+$ (U/mL)	TNF-α (U/mL)	IL-1 (U/mL)	IL-6 (U/mL)
Selenium (mg/kg)	Vitamin E (mg/ kg)	LPS						
0	0	0	343.83 ± 14.17	861.91 ± 26.62	792.45 ± 24.33	158.75 ± 9.48	23.4 ± 3.21	57.96 ± 3.46
		1	214.07 ± 12.35	241.87 ± 18.33	593.99 ± 15.67	48.07 ± 3.04	22.49 ± 0.94	35.68 ± 1.53
	50	0	270.69 ± 12.26	498.5 ± 19.61	869.66 ± 14.18	274.22 ± 6.63	18.35 ± 2.29	46.23 ± 3.01
		1	443.31 ± 10.1	541 ± 33.16	1479.51 ± 36.08	145.92 ± 11.85	22.05 ± 1.26	55.31 ± 2.8
	100	0	268.73 ± 28.07	1323.79 ± 24.17	560.93 ± 11.66	202.04 ± 10.93	20.88 ± 1.58	44.97 ± 2.92
		1	85.75 ± 4.12	654.63 ± 16.98	778.44 ± 12.02	95.76 ± 3.88	17.43 ± 2.54	25.2 ± 1.74
	200	0	276.46 ± 18.00	646.59 ± 26.64	994.17 ± 10.57	182.26 ± 9.58	21.7 ± 3.64	38.25 ± 1.52
		1	214.4 ± 13.06	1058.01 ± 33.9	848.62 ± 17.31	119.57 ± 3.78	21.6 ± 1.97	32.21 ± 3.65
0.3	0	0	231.02 ± 14.87	167.32 ± 13.44	494.2 ± 12.79	129.49 ± 11.53	15.93 ± 1.68	23.31 ± 3.54
		1	379.28 ± 19.86	572.52 ± 13.47	199.33 ± 18.58	228.24 ± 8.06	15.71 ± 2.41	26.46 ± 1.96
	50	0	488.32 ± 13.81	1520.98 ± 16.72	635.73 ± 26.18	151.39 ±14.91	17.01 ± 0.47	34.22 ± 3.71
		1	378.8 ± 24.04	662.2 ± 24.74	779.29 ± 16.85	233.87 ± 15.14	19.43 ± 2.23	64.42 ± 5.53
	100	0	386.43 ± 19.26	693.33 ± 24.26	1258.57 ± 36.5	220.68 ± 15.45	11.94 ± 0.78	48.2 ± 3.83
		1	394.81 ± 19.21	1283.33 ± 40.57	436.06 ± 16.08	270.24 ± 17.47	17.96 ± 2.52	43.72 ± 2.49
	200	0	233.71 ± 16.86	454.71 ± 48.89	965.92 ± 27.79	258.08 ± 23.31	17.9 ± 0.4	45.68 ± 2.09
		1	317.68 ± 11.51	683.39 ± 14.83	103.9 ± 13.81	365.95 ± 17.88	17.87 ± 1.15	36.42 ± 2.68
0.6	0	0	365.74 ± 21.82	540.2 ± 18.4	821.12 ± 17.11	363.62 ± 30.69	15.1 ± 4.17	65.02 ± 2.68
		1	269.83 ± 24.22	546.6 ± 17.61	496.95 ± 6.14	163.64 ± 2.1	12.97 ± 1.62	52.95 ± 1.96

50	0	334.25 ± 15.49	2159.75 ± 19.06	746.97 ± 22.14	454.13 ± 14.36	15.6 ± 1.43	66.16 ± 3.78
	1	353.23 ± 24.79	1753.87 ± 36.54	682.8 ± 13.34	237.19 ± 14.67	16.41 ± 0.04	35.95 ± 2.23
100	0	280.41 ± 19.31	845.63 ± 33.88	544.64 ± 9.29	184.82 ± 3.48	12.71 ± 0.49	28.31 ± 3.61
	1	186.31 ± 16.24	890.46 ± 15.28	1274.93 ± 14.54	173.12 ± 4.8	7.81 ± 0.13	28.12 ± 1.26
200	0	548.59 ± 19.1	1517.47 ± 24.61	1583.78 ± 17.31	342.86 ± 14.91	19.69 ± 0.36	67.44 ± 3.71
	1	551.81 ± 15.45	1270.4 ± 26.11	566.61 ± 17.86	365.37 ± 16.35	17.23 ± 1.66	47.76 ± 3.83

Each Value represents mean ± standard error (SE) of 3 individual broilers.
